# Supplementary material for: Chloroquine Inhibits Contraction Elicited by the Alpha-1 Adrenoceptor Agonist Phenylephrine in the Isolated Rat Aortas
Source: Int J Mol Sci. 2025 May 9;26(10):4556. doi: 10.3390/ijms26104556 (PMC12111159; doi:10.3390/ijms26104556)
Supplement: Supplementary file 1 [file ijms-26-04556-s001.zip › ijms-3585309-supplementary.pptx]

## Slide 1
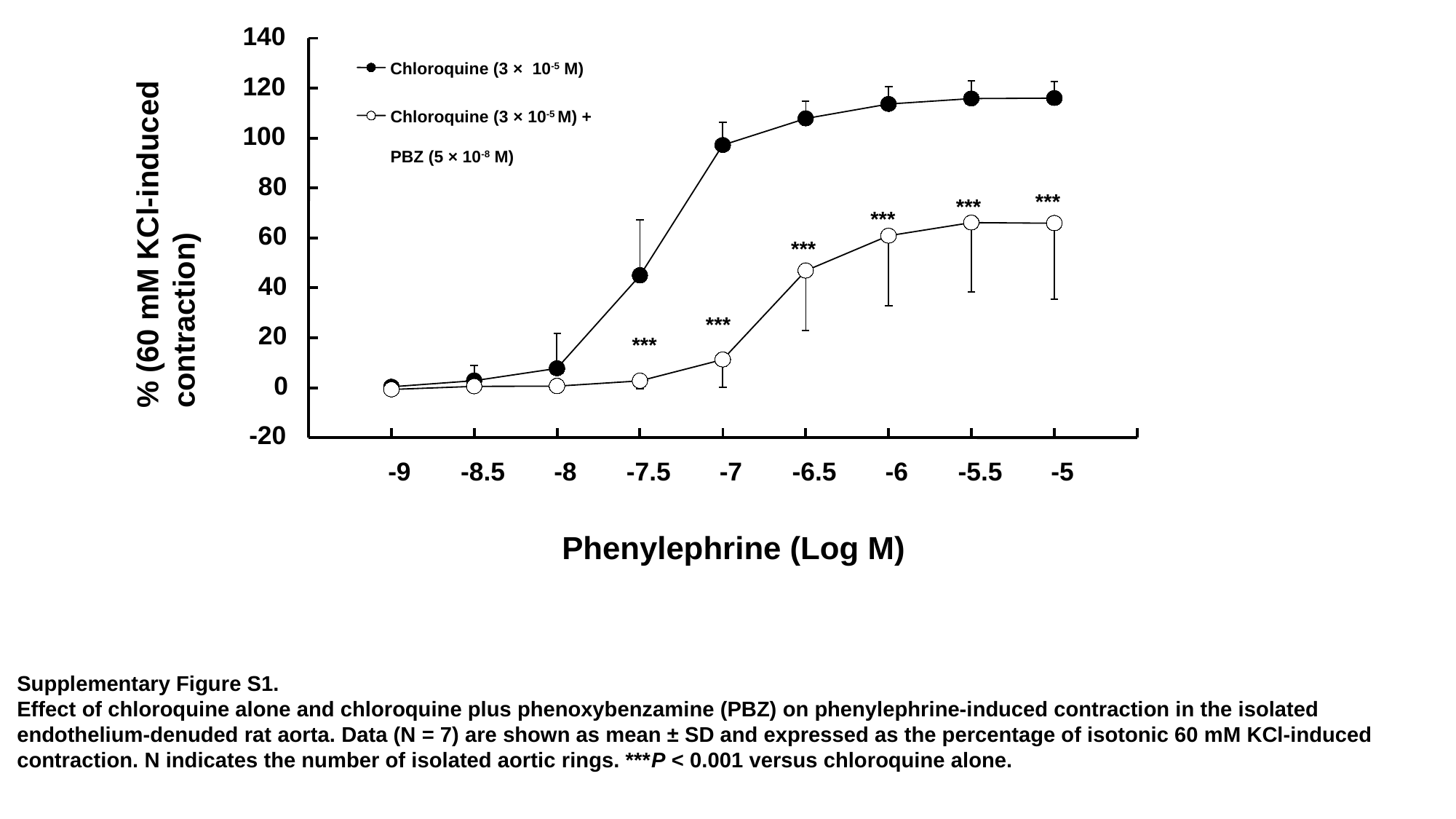

140
120
100
80
60
40
20
0
-20
-9
-8.5
-8
-7.5
-7
-6.5
-6
-5.5
-5
Chloroquine (3 × 10^-5^ M)
Chloroquine (3 × 10^-5^ M) +
PBZ (5 × 10^-8^ M)
***
***
% (60 mM KCl-induced
contraction)
***
***
***
***
Phenylephrine (Log M)
Supplementary Figure S1.
Effect of chloroquine alone and chloroquine plus phenoxybenzamine (PBZ) on phenylephrine-induced contraction in the isolated endothelium-denuded rat aorta. Data (N = 7) are shown as mean ± SD and expressed as the percentage of isotonic 60 mM KCl-induced contraction. N indicates the number of isolated aortic rings. ***P < 0.001 versus chloroquine alone.
